# The IKK Kinases: Operators of Antiviral Signaling

**DOI:** 10.3390/v2010055

**Published:** 2010-01-08

**Authors:** Alissa M. Pham, Benjamin R. tenOever

**Affiliations:** 1 Department of Microbiology, Mount Sinai School of Medicine, New York, NY 10029, USA; 2 Global Health and Emerging Pathogens Institute, New York, NY 10029, USA

**Keywords:** IKK, kinase, IKK-related, IKKalpha, IKKbeta, TBK1, IKKepsilon, NF-kappa B, interferon, antiviral, TLR, RIG-I, mda5, DAI

## Abstract

The ability of a cell to combat an intracellular pathogen requires a mechanism to recognize the threat and elicit a transcriptional response against it. In the context of virus infection, the cell must take measures to inhibit viral replication, meanwhile, convey warning signals to neighboring cells of the imminent threat. This immune response is predominantly mediated by the production of cytokines, notably, interferon beta (IFNβ). IFNβ signaling results in the transcriptional induction of over one hundred antiviral gene products whose timely expression renders infected cells more capable of inhibiting virus replication, while providing the uninfected cells with the reinforcements to generate a less permissive cellular environment. Induction of IFNβ and many aspects of the antiviral response pivot on the function of the IKK and IKK-related kinases. Despite sharing high levels of homology and some degree of functional redundancy, the classic IKK kinases: IKKα and IKKβ, and the IKK-related kinases: TBK1 and IKKɛ, perform distinct roles in regulating the host antiviral defense. These kinases serve as molecular operators in their cooperative ability to integrate incoming cellular cues and act on a range of essential antiviral transcription factors to reshape the cellular transcriptome during infection.

## 1. Introduction

The production of interferon beta (IFNβ) is a fundamental cellular response to combating pathogenic microorganisms. As such, the transcriptional induction of this gene has become a paradigm for virus-induced transcription. The signal transduction pathway responsible for IFNβ initiates with pathogen detection through both extra- and intra-cellular host pattern recognition receptors (PRRs) [1]. PRRs detect pathogen-associated molecular patterns (PAMPs) produced during replication. Binding of PRRs to their cognate ligands results in activation of downstream signaling molecules and the coordinated assembly of the enhanceosome, a multi-transcriptional complex responsible for recruiting RNA polymerase II to the transcriptional start site (TSS) of the IFNβ promoter [2].

The enhanceosome is composed of transcription factors (TFs) belonging to three distinct families: the interferon regulatory factors (IRFs), nuclear factor-kappa B (NF-κB) and specific members of the ATF and Jun families. Autocrine or paracrine signaling by IFNβ triggers the JAK/STAT pathway and the timely up-regulation of many IFN stimulated genes (ISGs) through the phosphorylation of signal transducers and activators of transcription 1 (STAT1) and STAT2, and the assembly of the Interferon Stimulated Gene Factor 3 (ISGF3) complex composed of STAT1, STAT2, and IRF9. These ISGs launch the cell, infected or uninfected, into an antiviral state, limiting virus replication while releasing chemokines to recruit immune cell reinforcements (For review see [3]).

Recognition of viral infection is mediated through three major pathways, Toll-like Receptors (TLRs), RIG-I-like Helicases (RLHs), or through intracellular DNA receptors [1,4,5]. While each sensory pathway utilizes distinct adaptor proteins to relay downstream signals, many of these events converge at the level of the IκB (IKK) and IKK-related kinases [6]. The IKK kinases, referred to as the classical IKKs, include IKKα and IKKβ and are responsible for the activation of NF-κB [7]. The IKK-related kinases, which include TBK1 (also called NAK and T2K) and IKKɛ (also called IKK-i), were recently identified as the *bone fide* kinases for IRF3 and IRF7 [8,9].

As evident by the high sequence similarity and *in vitro* substrate specificities of IKKα and IKKβ, as well as that of TBK1 and IKKɛ, there is a significant amount of functional redundancy between the kinases. Despite their commonalities, knock-out studies of each kinase member has also demonstrated additional diverse functions that shape the cellular response to a wide range of environmental cues [7]. In general, the functional relationship of IKKɛ and TBK1 is akin to that of IKKα and IKKβ. Whereas TBK1 and IKKβ are necessary for IRF3/7 and NF-κB activation, respectively, IKKɛ and IKKα are sufficient to induce these pathways, but their absence does not disrupt transcriptional induction. Moreover, each of the kinases has also been associated with unique non-canonical antiviral signaling pathways. This review focuses strictly on the roles these kinases play in the cell-autonomous innate immune signaling, and seeks to clarify what is currently understood concerning the upstream activation of the individual kinases and their substrates following virus recognition.

## 2. Transcriptional regulation of the IFNβ promoter

The expression of IFNβ is a metabolically expensive process, resulting in broad transcriptional effects that inhibit many vital cellular processes. Evidently, the production of IFNβ is a tightly regulated event, involving the activation and cooperation of multiple enzymes and adaptors following virus recognition. Extensive research has been done on its structural composition and its mode of activation [2]. The IFNβ promoter is made up of four positive regulatory domains (PRDIV, III, I and II with respect to the TSS), which are bound by ATF2/c-Jun, two heterodimers of IRF3/7, and NF-κB respectively, forming a multi-subunit complex called the enhanceosome [10,11]. Assembly of the enhanceosome requires the coordinated activation of all three TF families, as it is largely mediated by cooperative binding [2]. As each of the PRD elements deviate from the TF core consensus sites, assembly of the enhanceosome is believed to begin with ATF2/c-Jun followed by an IRF3/7 heterodimer on PRDIV and PRDIII, respectively. Although PRDIII is an imperfect IRF binding element, the interaction between ATF2/c-Jun and the IRFs provide the additional stability necessary to maintain DNA contacts [11,12]. Cooperative binding of PRDIV and III, in turn, support occupancy on PRDI with another IRF3/7 heterodimeric complex, as well as the binding of a p50/p65 heterodimer of NF-κB on PRDII [10]. As ATF2 and c-Jun are activated in response to a wide variety of stress-related stimuli that occur following virus infection, assembly of the enhanceosome is largely dependent on NF-κB and IRF3/7, and therefore, the catalytic activity of the IKK and IKK-related kinases.

The NF-κB family of TFs regulates a wide array of genes involved in immunity, inflammation, and cell growth [13]. The NF-κB family consists of five members: RelA(p65), RelB, c-Rel, p105, and p100, whose subunits form homodimers or heterodimers that, upon activation, enter the nucleus to bind upstream of target genes [14]. Under unstimulated conditions, latent p50 and p65 dimers, the most studied TFs in the NF-κB pathway, are maintained in the cytoplasm by a family of IκB proteins, most notably IκBα. Upon PRR stimulation, a large multi-subunit complex containing IKKα, IKKβ, and NEMO (also called IKKγ), phosphorylates the IκB proteins on serine 32 (S32) and S36, resulting in subsequent polyubiquitination of lysines 21 and 22, targeting them for degradation [15]. Loss of IκB liberates NF-κB dimers, exposing their nuclear localization sequence and allowing them to translocate into the nucleus [15].

While NF-κB is activated by a variety of stimuli, the IRFs are primarily known for their roles in anti-microbial defense. They were first identified based on their ability to bind elements upstream of the IFNβ promoter [16]. The IRF family is comprised of nine members whose functions are implicated in various cell processes including cell growth, cytokine signaling, and most notably, pathogen response [17]. In the cellular response to virus infection, IRF3 and IRF7 play a predominant role in the transcriptional induction of the type I IFN (IFN-I) family, comprised of a single IFNβ gene and a cluster of IFNα genes [18]. While IRF3 is expressed ubiquitously, basal expression of IRF7 is kept at low levels in most cells and is induced after the initial phase of IFNβ expression. This is followed by the induction of the IFNα subtypes, which are induced solely by IRF7 homodimers [18]. The low basal expression of IRF7 is thought to account for the stochastic expression of IFNβ following virus infection, a phenomenon that does not occur in cells primed with IFN-I [19]. Plasmacytoid dendritic cells, however, maintain high levels of basal IRF7 and are capable of producing abundant amounts of IFN-I in response to infection. IRF3 and IRF7 activation is mediated by a wide range of phosphorylation events, many of which are directly induced by the IKK-related kinases to coordinate protein dimerization, nuclear translocation, DNA binding, and the induction of IFNβ [8,20–24].

## 3. The IKK complex

**Figure 1.**
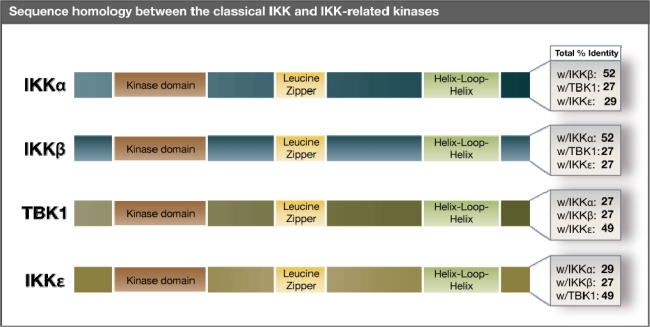
Amino acid sequence homology of the classical IKK and IKK-related kinases. Amino acid sequence comparisons between human IKKα, IKKβ, TBK1, and IKKɛ.

The classical IKK kinases, IKKα and IKKβ, are essential for the activation of NF-κB TFs in response to diverse stimuli [25]. Together with NEMO, they form a multi-subunit IKK complex that collectively function to phosphorylate IκB and release NF-κB, resulting in its nuclear translocation [26,27]. The IKK kinases share 52% amino acid identity [28], each having molecular weights of approximately 80–90 kDa (Figure 1). As structurally similar proteins, they share a protein kinase domain in the N-terminus followed by leucine zipper (LZ) and helix-loop-helix (HLH) motifs at the C-terminus [25]. NEMO is approximately 50 kDa in size, containing two coiled-coil domains along with LZ and zinc finger motifs. Whereas IKKα and IKKβ are responsible for the catalytic activity of the complex, NEMO does not perform any enzymatic functions, but serves as a regulatory hub [29]. While the three individual subunits together form a complex of 210 kDa, the purified IKK complex is estimated to be 700–900 kDa in size. Hence, it is believed that the IKK complex is comprised of multiple oligomeric forms of each protein, which may confer aspects of substrate specificity [30,31].

The exact role of IKKα and IKKβ as they pertain to inducing IκBα degradation, and their responsibilities within the IKK complex during virus infection, still remains somewhat unclear. To assess the relative contributions of each protein, studies have focused on using inactive forms of each kinase or through traditional knock-out murine fibroblast cell lines. Results from such studies have suggested that there are, in fact, distinctions between the two kinases. While IKKβ plays a predominate role in catalyzing IκBα degradation in response to pro-inflammatory stimuli [32], more recent studies have suggested a number of alternative IKKα -dependent pathways that do not involve IKKβ or the degradation of IκB proteins [33–35]. IKKα has been found to mediate the processing of the NF-κB p100 subunit from its latent form into an active conformation [33,34]. In addition, IKKα has also been described to be a *bona fide* kinase of IRF7 following TLR7 stimulation in conjunction with IL-I receptor-associated kinase-1 (IRAK1) and IRAK4 [35]. Alternative roles for IKKβ have also been described, including a recent study that highlights a role for IKKβ in the expression of certain IFNγ-dependent genes outside of NF-κB activation [36]. In addition, research in knock-out fibroblasts have demonstrated that a complete loss of known NF-κB TFs does not significantly impair virus-induced IFNβ, suggesting other compensatory factors can be activated by the IKK kinases [37]. These alternative pathways demonstrate both the redundancy and unique signaling functions these kinases perform in tuning the cellular antiviral transcriptome.

Despite IKKα’s role in the non-canonical NF-κB pathway, its function in the IKK complex is still essential for the induction of many pro-inflammatory cytokines. Studies with IKKα-deficient cells display defects in the expression of a subset of NF-κB-dependent genes [38]. This defect was fully rescued when wild type IKKα was reconstituted in *Ikbka^−/−^* cells. Furthermore, rescue experiments were also performed using a kinase-inactive form that was able to rescue the expression of 28% of the genes whose expression was lost. Interestingly, this data imparts a role for IKKα in maintaining basal expression of certain NF-κB-dependent genes as well as those that are expressed in response to stimuli.

## 4. The IKK-related kinases

Like the classical IKK kinases, the IKK-related kinases appear to demonstrate both redundant and unique functions aimed at shaping the cellular response to virus infection. Although it was initially thought that the functional redundancy in activating NF-κB may extend to all four members of the IKK family, the groups of Hiscott and Maniatis demonstrated that the IKK-related kinases were responsible for the phosphorylation and activation of IRF3/7 in response to virus infection [8,9].

TBK1 and IKKɛ were identified earlier in separate screens searching for IKKα/IKKβ-homologous genes and lipopolysaccharide (LPS)-inducible genes, respectively [39,40]. The kinase domains of TBK1 and IKKɛ share greater than 70% amino acid sequence identity but are widely divergent at their C-terminus, resulting in an overall homology of less than 50% (Figure 1). Comparable to IKKα and IKKβ, TBK1 and IKKɛ also share an N-terminal kinase domain followed by C-terminal LZ and HLH motifs which results in an overall homology of approximately 30% between the IKK and IKK-related kinases (Figure 1). This structural and sequence similarity also confers limited substrate overlap as each of these kinases can phosphorylate S36 of IκBα, one of the two critical serines involved in its degradation [41].

Despite their limited involvement in the NF-κB pathway, continued studies on the IKK-related kinases strongly suggest that TBK1 and IKKɛ serve primary roles in antiviral signaling as activators of IRF3/7 [42,43]. The finding that both TBK1 and IKKɛ reconstitute complete C-terminal phosphorylation of the critical residues in IRF3/7, both *ex vivo* and *in vitro,* has further supported this notion. However, despite the apparent redundancy between the IKK-related kinases, knock-out experiments suggest that TBK1, like IKKβ and its role in NF-κB activation, serves as the primary inducer of IRF3/7. Although *Tbk1^−/−^* mice are embryonic lethal, cells derived *in utero* demonstrate a complete loss in IRF3 phosphorylation and induction of IFN-I [9,44]. Conversely, genetic disruption of *Ikbke* (the gene which encodes IKKɛ) does not demonstrate any defects in IFNβ induction, either *in vivo* or *ex vivo* [45]. However, as IKKɛ expression in most cell types requires virus-induction, IKKɛ likely plays a redundant role to TBK1 during the late stages of virus infection [46]. This idea is supported by the fact that expression of IKKɛ can rescue IFNβ inducibility in response to virus infection in *Tbk1^−/−^* fibroblasts and bone marrow-derived macrophages [43]. In contrast to IFN-I induction, mice lacking IKKɛ demonstrated a deficiency in an IFN-related function that is distinct from IRF3/7 activation. Similar to the unique and complementing functions of IKKα, IKKɛ was found to influence the binding specificity of ISGF3 in an IFNβ-dependent manner [45]. In addition, IKKɛ has been reported to decrease the activity of CYLD, a deubiquitinating enzyme that negatively regulates IRF3/7 activation, thereby indirectly increasing IRF-mediated signaling [47,48]. Furthermore, IKKɛ has been found to be important for direct phosphorylation of specific NF-κB subunits and in the induction of a specific subset of pro-inflammatory cytokines through the CCAAT/enhancer-binding protein TFs [49–51]. Altogether, these studies reveal distinct roles for IKKɛ in innate immunity, and are examples of how multiple signal transduction pathways can be integrated to customize an immune response.

## 5. Detection of virus infection

**Figure 2.**
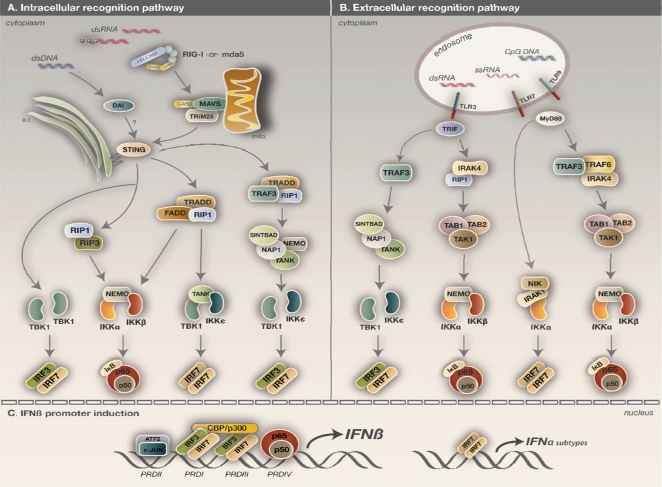
Induction of type I IFN by intracellular and extracellular virus recognition pathways. **A**. Recognition of intracellular PAMPs is mediated by the RNA helicases, RIG-I and mda5, and DNA sensor, DAI. RIG-I and mda5 both detect distinct forms of dsRNA. RIG-I preferentially binds dsRNA containing 5′-triphosphates, whereas mda5 binds longer blunt-end dsRNA molecules. Downstream signaling via RIG-I/mda5 requires interactions with the mitochondrial-associated adaptor, MAVS, mediated by the CARD domains found on each molecule. Following ubiquitination by TRIM25 and possible association with an ER-associated factor called STING, RIG-I transmits signals downstream to distinct TRADD-containing complexes for TBK1/IKKɛ and IKKα/IKKβ activation. Activation of the IKK and IKK-related kinases results in IRF and NF-κB phosphorylation, resulting in their nuclear translocation. DAI is a recently identified PRR that detects B-form DNA found in the cytosol and induces IFN-I production through IRF3 and NF-κB activation. This mode of activation requires further investigation. However, DAI-mediated activation of IRF3 is known to require TBK1, whereas RIP1 and RIP3 are necessary for NF-κB activation. **B**. TLRs are expressed primarily in macrophages and DCs, and sense PAMPs found in the extracellular environment. TLR3 detects dsRNA and coordinates IRF and NF-κB activation through adaptor, TRIF. TLR7 and TLR9 sense ssRNA and CpG motifs of microbial DNA, respectively, and unlike TLR3, they utilize MyD88 to signal downstream. **C**. Induction of IFN-I requires the activity of the IKK and IKK-related kinases for the activation and nuclear translocation of important IRF and NF-κB TFs. The IFNβ promoter contains four PRD domains that are occupied by ATF2/c-JUN, two IRF3/IRF7 heterodimers, and p50/p65. Cooperative binding of these TFs with histone remodeling factors, CBP and p300, together form an enhanceosome that drives IFNβ production.

Recognition of virus infection warrants an effective immune response to clear the host of invading pathogens. Detection of microbial invasion can derive from both extra- and intra-cellular PAMP sources. The viral PRRs responsible for extracellular ligand binding encompass members of the TLR family. Intracellular viral PRRs represent a broad range of sensors that include: retinoic acid inducible gene (RIG-I) and melanoma differentiation associated gene 5 (mda5) which recognize foreign RNA [1]. In addition to RNA sensors, the cell is also equipped with a number of cytoplasmic DNA sensors [4,5]. Upon encountering their cognate ligand, each pathway employs different adaptor molecules to signal downstream and prompt IFN-I production. All three pathogen recognition pathways require the catalytic activity of the IKK and IKK-related kinases in order to activate NF-κB and IRF3/7 TFs (Figure 2).

### 5.1. IKK activation via the TLR pathway

Extracellular or endosomal recognition of virus infection requires detection by the TLR family of membrane proteins. Of the 12 members of the TLR family (TLRs 1–12), TLR3, 7, 8, and 9 are predominantly responsible for viral recognition [52]. While TLR3 detects double-stranded RNA (dsRNA), a common by-product of virus replication or defective interfering particles, TLRs 7 and 8 detect single-stranded RNA found in the genomes of certain viruses and from necrotic cells [1]. TLR9, on the other hand, recognizes un-methylated CpG motifs that are characteristic of microbial nucleic acids [52]. Unlike the cytosolic PRRs, TLRs are expressed primarily by immune cells, such as macrophages and dendritic cells (DCs), but can also be transcriptionally up-regulated in non-myeloid cells following virus infection. Upon activation, TLRs relay downstream signals via their intracellular Toll/Interleukin-1 receptor (TIR) domains, which bind either myeloid differentiation factor 88 (MyD88) or TIR-domain-containing adaptor-inducing IFNβ (TRIF) to recruit additional intracellular adaptors. All TLRs except for TLR3, which signals through TRIF, utilize MyD88 [52].

MyD88-dependent activation of NF-κB involves signaling via TRAF6, which upon TLR activation, is recruited by members of the IRAK family: IRAK1, 2, and 4 [53,54]. Phosphorylation events between the IRAKs, lead to their activation and subsequent recruitment of TAK1/TAB1/TAB2 [53]. Activated TAK1 phosphorylates the IKK complex containing IKKα, IKKβ, and NEMO. As a result, IκB proteins are targeted for phosphorylation and degradation, allowing NF-κB activation and nuclear translocation [7].

MyD88-mediated activation of the IRFs appears to be selective for IRF7 activation and occur independently of the IKK-related kinases [55]. Upon TLR7 activation, a complex containing MyD88, IRAK1 and IKKα is recruited to mediate the phosphorylation and subsequent activation of IRF7 [34]. TLRs that utilize MyD88 display defects in IFN-I production in cells lacking IRF7 [56]. As macrophages and DCs express high basal levels of this critical transcription factor, this distinct signaling pathway contributes to their ability to be potent producers of IFN-I. Separate studies suggest NF-κB-inducing kinase (NIK) may also be a member of this complex [57].

TRIF-dependent activation of NF-κB via TLR3 occurs distinctly from the MyD88 pathway. Activation of NF-κB is dictated by IRAK4 and receptor interacting protein 1 (RIP1), which then relay signals to the TAK1 complex, resulting in IKKα/IKKβ activation [58]. Unlike the MyD88 pathway, TRIF signaling does not seem to require TRAF6 [59], although IRAK-independent NF-κB activation has been observed [60]. Similar to the MyD88 pathway, TLR3-mediated activation of IRF3 stems from the TRAF3 adaptor, which forms a multimeric complex with TBK1 and IKKɛ [61], and possibly other recently identified adaptors, SINTBAD and NAP1, which are discussed below [62].

### 5.2. IKK activation via the RLH pathway

In addition to extracellular PAMP recognition, cells have TLR-independent mechanisms of detecting viral nucleic acids generated in the cytoplasm. The RNA helicases, RIG-I and mda5, are similar in structure, both having a caspase recruitment (CARD) domain at the N-terminus and a DEx(D/H) box RNA helicase domain at the C-terminus. Initial studies suggested that RIG-I and mda5 performed redundant roles in sensing virus infection. However, ongoing studies continue to provide more insight into their specificity for certain motifs [63]. While both helicases respond to viral RNA in the cytoplasm, RIG-I preferentially binds short dsRNA molecules with exposed 5′ tri-phosphates, whereas mda5 binds longer blunt-end dsRNA [64,65]. Stimulation of RIG-I and mda5 causes a conformational change in their CARD domains, leading to the recruitment of a downstream CARD-containing adaptor protein called mitochondrial antiviral signaling protein (MAVS) (also referred to as Cardif, IPS-1, and VISA) [66–69]. As the name suggests, MAVS is localized at the outer mitochondrial membrane via its transmembrane domain, and serves as a platform for additional factors to bind and transmit upstream signals. The interaction between RIG-I and MAVS requires ubiquitination of the first CARD domain of RIG-I by tripartite motif 25 (TRIM25) [70]. It remains unknown whether ubiquitination is also required for mda5 function.

Downstream signaling via the RLH pathway is mediated by MAVS and its interaction with multiple effector complexes. Initial characterization of this signal transduction pathway implicated TRAF6 in the activation of NF-κB and ATF2/cJun, while TRAF3 was found to recruit TBK1 for subsequent IRF3 phosphorylation [71]. Ongoing studies in this area continue to uncover novel components of this cascade, and have suggested that TRAF6 may play a more significant role in TLR-mediated activation of the IKK kinases whereas TRAF3 remains crucial for TBK1 and IKKɛ activation [72]. RLH-dependent activation of the IKK kinases is now thought to occur through Fas-associated death domain-containing (FADD) protein and TNFRSF1A-associated via death domain (TRADD), which are both required for RIG-I/mda5-mediated, but not TLR-mediated, antiviral signaling [72]. Experiments with *Fadd^−/−^* MEFs demonstrate impaired NF-κB and IRF activation in response to dsRNA [72]. Similarly, defects in IFN-I responses were also observed for the adaptor, RIP1. While a direct interaction with MAVS has not been described for FADD, TRADD has been shown to directly bind MAVS [72]. Based on this current model, it can be envisaged, that upon RIG-I activation, MAVS recruits TRADD to orchestrate the formation of a multimeric complex containing FADD, RIP1, and TANK that can then dictate NF-κB and IRF activation [73]. A role for caspase 8 and caspase 10 has also been implicated in FADD-dependent activation of the IKK kinases [74]. Furthermore, a separate study with *Fadd^−/−^* cells reported its involvement in the regulation of IRF7 homodimers for secondary IFNα production. *Fadd^−/−^* MEFs displayed defects in production of IFNα subsets that can be rescued by exogenous expression of IRF7 [75]. As described for the TLR pathway, TRAF3 also participates in the RLH pathway to trigger TBK1 and IKKɛ activation. Similar to the classical IKK-NF-κB activation, TBK1/IKKɛ activation is also thought to involve a complex consisting of TRADD and RIP1 [73].

Ongoing studies have recently implicated roles for cellular organelles in innate immunity [6,76]. Although MAVS, which is localized to the mitochondria, does not interact directly with TBK1 and IKKɛ, a population of IKKɛ has also been found to associate with this organelle [77]. In contrast, TBK1 localization is distributed throughout the cytoplasm [78]. Furthermore, NLRX1, a protein localized in the mitochondrial membrane, is known to interact with MAVs and to negatively regulate the RLH pathway [79]. An antiviral role has also been described for the endoplasmic reticulum (ER), in which a newly identified protein, STING, is known to bind and modulate antiviral responses (Its function will be discussed in the following section). Taken together, it appears the innate immune response to virus infection is a complex operation requiring the timely recruitment and activation of multiple components. Therefore, employing cellular organelles as platforms for signal transduction may ensure efficiency and specificity for the cell to react to various cues.

### 5.3. IKK activation via the DNA sensory pathway

Sensory mechanisms for detecting cytoplasmic DNA are just recently being characterized [4,5,80–83]. DNA-dependent activator of IFN regulatory factors (DAI) was the first identified sensor of cytoplasmic dsDNA [5]. Also known as ZBP1 and DLM-1, DAI is IFN-I-inducible and ubiquitously expressed in a variety of cell types. It shares DNA-binding domains similar to adenosine deaminase activating on RNA1 (ADAR1), a known ISG with antiviral activity [45]. Upon engagement with its ligand, DAI undergoes conformational changes to recruit and activate TBK1 and IRF3 for IFNβ production. To activate NF-κB, DAI interacts with the kinases RIPI and RIP3, via its RIP homotypic interacting motif (RHIM) domain [80,84]. Furthermore, its been demonstrated that TBK1 can target DAI for phosphorylation, an event thought to increase DAI’s affinity for TBK1 and IRF3, and thus amplify IRF3 activation and nuclear translocation. Details concerning the formation of this complex and whether other adaptor proteins are involved require further investigation. A recent study sought to examine whether IKKɛ contributes to IRF3 activation in response to the DNA sensory pathway. *Tbk1^−/−^* and *Ikbke^−/−^* fibroblasts were reconstituted with equivalent amounts of TBK1 and IKKɛ to examine the relative contributions of each kinase. Results showed TBK1 was better capable of rescuing IFNβ induction than IKKɛ [85]. Thus, similar to RLH-mediated induction, IFNβ transcription is initiated primarily by TBK1 in response to intracellular DNA detection.

Following the discovery of DAI, Ishikawa *et al*. uncovered a novel protein referred to as stimulator of interferon genes (STING) that was shown to induce activation of IFNβ and NF-κB promoters in response to cytosolic DNA [4]. *Sting^−/−^* MEFs displayed defective induction of IFNβ in response to transfected B-form DNA, infection by DNA virus, herpes simplex virus 1, and vesicular stomatitis virus, implicating its role in IFNβ induction in response to both microbial DNA and RNA [4]. Interestingly, STING contains five transmembrane regions that associate with the ER, and this attachment is required for proper signaling [4]. Co-immunoprecipitation studies suggest STING interacts with both RIG-I and TBK1 to modulate innate immune signaling. However, STING does not appear to mediate signals downstream of mda5. Although STING is required for antiviral responses against foreign intracellular DNA, the mechanism and upstream components involved remain unidentified.

Lastly, cytosplasmic DNA detection has also been found to induce activation of the “inflammasome”, an NF-κB-dependent pathway that controls the catalytic cleavage of IL1β and IL18 [86]. In this pathway, NF-κB activation occurs in a caspase 1- and 3-dependent manner involving AIM2 (absent in melanoma 2), a member of the IFN-inducible HIN-200 family [81–83]. Although the exact molecular mechanisms leading to activation of the IKK-kinases remains unknown, the AIM2-dependent activation is thought to involve its oligomerization following cytoplasmic DNA detection to induce the subsequent activation of PYD and CARD domain-containing protein PYCARD (also known as ASC). NALP3, also known as NLRP3, has also been implicated in this signaling pathway [86]. The roles, if any, of the IKK-related kinases in response to AIM2 signaling remain unknown.

### 5.4. IKK activation via IFN signaling

In addition to kinase activation in response to direct virus infection or PAMP stimulation, IKKβ and IKKɛ can be activated directly in response to IFNγ and IFNβ, respectively [45,87]. Although the molecular details regarding kinase activation remain somewhat unclear for these pathways, loss of kinase expression, in each case, reduced an important subset of genes implicated in antiviral signaling. In addition, IFN-mediated activation of both IKKβ and IKKɛ induced a transcriptional response that was independent of NF-κB and IRF3/7, respectively. While known to be IKKα-independent, the pathway implicated in IFNγ-mediated induction of IKKβ remains to be elucidated [35]. With regards to IKKɛ, IFNβ signaling results in the direct phosphorylation of IKKɛ at a PI3K consensus site. As IFNβ-mediated activation of PI3K has already been characterized [88], the upstream events leading to phosphorylation are likely to be shared. IKKɛ activation by IFNβ induces the subsequent phosphorylation of STAT1, which changes the binding preferences of ISGF3, and is responsible for the transcriptional induction of a broader range of ISGs [45].

## 6. The IKK-related kinases and their adaptors

Although many studies have confirmed the involvement of TBK1 and IKKɛ in IRF3/7 phosphorylation, details concerning upstream activation remain complicated and elusive. Ongoing studies on TBK1 and IKKɛ continue to reveal novel interacting partners that are required for proper IFNβ induction in response to various stimuli. These include TRAF -associated NF-κB activator (TANK), NAK-associated protein 1 (NAP1), and similar to NAP1 TBK1 adaptor (SINTBAD) [30]. Adaptors or scaffold proteins do not perform any catalytic activity, but are, nonetheless, critical to various biological functions. Similar to the IKK kinases that require the adaptor NEMO to mediate their catalytic activity, studies suggest there exists an analogous adaptor for TBK1 and IKKɛ which may include NEMO itself [78]. The first to be characterized was TANK, which was initially identified as a TRAF2-binding protein involved in the activation of NF-κB-dependent genes. It was later shown that TANK directly binds TBK1 and IKKɛ constitutively at its N-terminus, and is perhaps involved in mediating the events leading to IRF3/7 activation [62,89]. Although TANK is required for TBK1/IKKɛ-mediated IRF3/7 activation in response to LPS (mediated by TLR4), it has been recently shown TANK is dispensable in the TLR3 pathway [90].

NAP1 is another TBK1/IKKɛ-interacting protein that has also been implicated in NF-κB activation following TNFα stimulation [91]. NAP1’s association with TBK1 and IKKɛ suggests it may also mediate the assembly of a TBK1/IKKɛ complex that could drive IRF3/7 activation. The most recently identified candidate, SINTBAD, shares a conserved TBK1/IKKɛ binding domain with TANK and NAP1 [62]. RNA interference against SINTBAD was used to demonstrate impaired IRF activation in response to Sendai virus infection. Based on the current studies, it seems TANK, NAP1, and SINTBAD perform similar functions in terms of facilitating TBK1/IKKɛ recruitment and activation. They all share similar structural properties that suggest they may perform redundant roles. However, knock-down studies have implicated the requirement of each of these proteins in producing a potent antiviral response. Furthermore, the requirement for NEMO in both IKK and IKK-related kinase mediated signaling suggests the presence of a massive signaling complex comprised of all four IKK kinases whose function is dictated by the specific adaptors recruited [78]. Further studies are of course needed to determine what kind of complexes are formed between TBK1/IKKɛ and the adaptors mentioned above, whether all three adaptors are involved, or whether distinct complexes are formed in response to different stimuli.

## Conclusion

Concerted efforts to study the cellular response to virus infection have dramatically advanced our understanding of viral detection, cell signaling, and the production and function of an array of cytokines. Coordinating these events are four very important kinases, which perform both general and refined functions to ensure that the resulting transcriptional response is perfectly tailored to inhibit virus replication. These events require integration of a wide range of signaling cues and the subsequent coordinated activation of select TFs making each of them molecular operators of antiviral signaling.
